# Reaction time and brake pedal force after total knee replacement: timeframe for return to car driving

**DOI:** 10.1007/s00167-020-06105-2

**Published:** 2020-06-24

**Authors:** Stephanie Kirschbaum, Michael Fuchs, Marion Otto, Clemens Gwinner, Carsten Perka, Ufuk Sentürk, Tilman Pfitzner

**Affiliations:** 1 Center for Musculoskeletal Surgery, Charité-University Hospital Berlin, Charitéplatz 1, 10117 Berlin, Germany; 2 Department of Orthopedics, RKU University Hospital Ulm, Ulm, Germany; 3 Department for Musculoskeletal Surgery, Vivantes Hospital Spandau, Berlin, Germany

**Keywords:** Brake reaction time, Brake pedal force, Braking force, Car driving, Total knee arthroplasty, Total knee replacement

## Abstract

**Purpose:**

This prospective cohort study aimed to examine objective and subjective parameters in patients who underwent total knee replacement (TKR) to assess from when on driving a car can be deemed safe again.

**Methods:**

Thirty patients (16 women, 14 men, age 66 ± 11 years) who received TKR of the right knee and 45 healthy controls (26 women, 19 men, age 32 ± 9 years) were asked to perform an emergency braking manoeuvre using a driving simulator. Brake pedal force (BPF), neuronal reaction time (NRT), brake reaction time (BRT), and subjective parameters (pain, subjective driving ability) were measured preoperatively as well as 5 days, 3–4, and 6 weeks after TKR.

**Results:**

Preoperative NRT was 506 ± 162 ms, BRT 985 ± 356 ms, and BPF 614 ± 292 N. NRT increased to 561 ± 218 ms, BRT to 1091 ± 404 ms and BPF decreased to 411 ± 191 N 5 days after TKR. Three weeks after surgery, NRT was 581 ± 164 ms and BRT 1013 ± 260 ms, while BPF increased to 555 ± 200 N. Only BPF showed significant differences (*p* < 0.01). In week 6, all parameters were restored to baseline levels; patients showed significant pain decrease and evaluated their driving ability as “good” again.

**Conclusion:**

BPF was the only parameter displaying a significant postoperative decrease. However, preoperative patients’ baseline levels and subjective confidence in driving ability were only reached 6 weeks after the operation. These results indicate that a minimum waiting period of 6 weeks should be considered before patients can safely participate in road traffic at their individual preoperative safety level again.

**Level of evidence:**

II.

## Introduction

For many patients, driving a car is essential and grants them mobility, flexibility and independence and is usually compromised due to pain, use of walking aids and partial weight bearing after lower limb surgery. In clinical practice, patients, therefore, frequently inquire about recovery times and when they can safely drive again after total knee replacement (TKR). These questions typically remain unanswered as there are no standardised recommendations.

Performing a sufficient emergency braking is one of the most relevant skills to drive a car safely [9, 18]. In particular, neuronal reaction time (NRT), brake reaction time (BRT) [15, 19] and brake pedal force (BPF) [17] are the most relevant parameters of an effective braking process. Current recommendations for a save return to car driving after lower limb surgery range from 10 days to 8 weeks and are based on a small number of heterogeneous studies merely investigate the change in reaction times [1, 8, 11, 12, 14, 16]. However, reaction time alone is inadequate to obtain a qualitative and quantitative assessment of the braking process, as it only describes the speed but does not represent the quality and strength of the braking process itself. Beside reaction time, BPF is essential for performing sufficient emergency braking manoeuvres. Although BPF after lower limb surgery was investigated in some studies before, there is very little and no recent data concerning BPF after modern TKR [13, 18].

So far, no other study evaluates the ability of car driving as combination of reaction time, BPF and subjective outcome parameters following TKR. The purpose of this study was to investigate objective and subjective safety parameters after TKR to evaluate safe return to car driving. It was hypothesised that car driving ability will not be restored before 6th postoperative week.

## Materials and methods

### Study design

Braking behaviour was evaluated in a healthy control group (*n* = 45, 26 women, 19 men, age 32.1 ± 8.8 years [21–56], BMI 22.5 ± 4.2 kg/m^2^) and in a patient group (*n* = 30, 16 women, 14 men, age 66.3 ± 10.7 years [47–88], BMI 31.7 ± 7.5 kg/m^2^). All individuals in the patient group had undergone TKR of their right knee joint. All study participants possessed a valid class B driving license and at least 2 years of regular driving experience (min. 0.5×/week). 64.4% of the control group subjects (*n* = 29) drove a car on an almost daily basis, 17.8% (*n* = 8) drove a motor vehicle at least 1–2 × per week and 17.8% (*n* = 8) drove at irregular intervals (0.5 × per week). Fifty percent (*n* = 15) of the TKR patients drove a car on an almost daily basis, 20% (*n* = 6) drove a motor vehicle at least 1–2 × per week and 30% (*n* = 9) drove at irregular intervals (0.5 × per week). There was no significant difference in driving frequency between the control and patient groups.

**Fig. 1:**
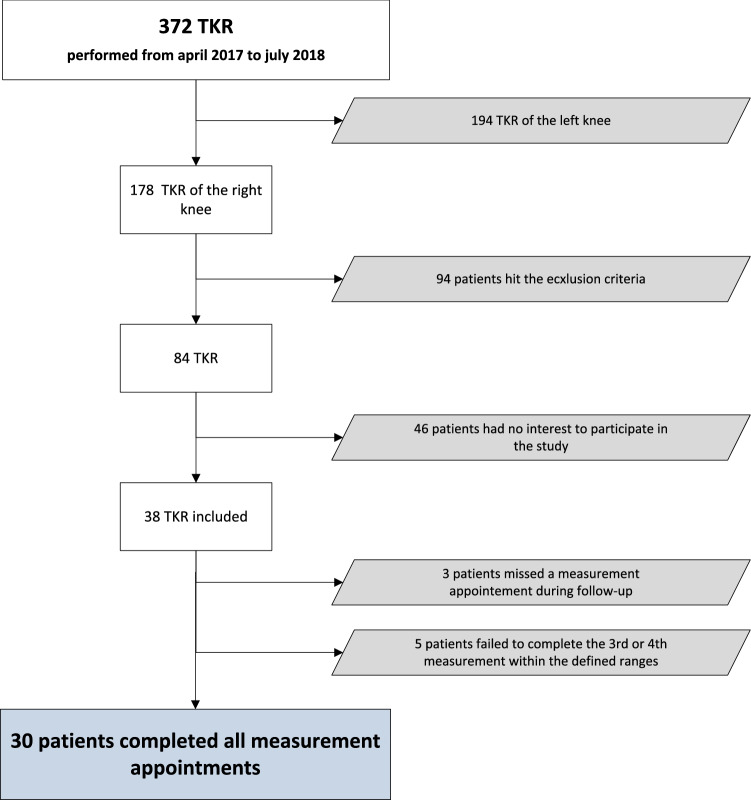
Flow chart of patient enrolment; 46 patients declined to participate for various reasons (distance, fear, no interest), 38 agreed. Three patients missed a follow-up appointment and 5 patients failed to complete the 3rd or 4th measurement within the defined ranges and were, therefore, excluded from this study. Thirty patients remained enrolled. *TKR* total knee replacement

Patients with chronic lower limb symptoms (apart from in the operated knee joint) and with chronic upper limb or back pain were excluded from the study, as were patients who had missed one follow-up appointment. Every patient fulfilling the inclusion criteria was invited to participate in the study. Patient enrolment and allocation is portrayed in Fig. 1. Informed consent was obtained from each participant prior to enrolment in this study.

### Imitation of the braking process in the driving simulator

**Fig. 2:**
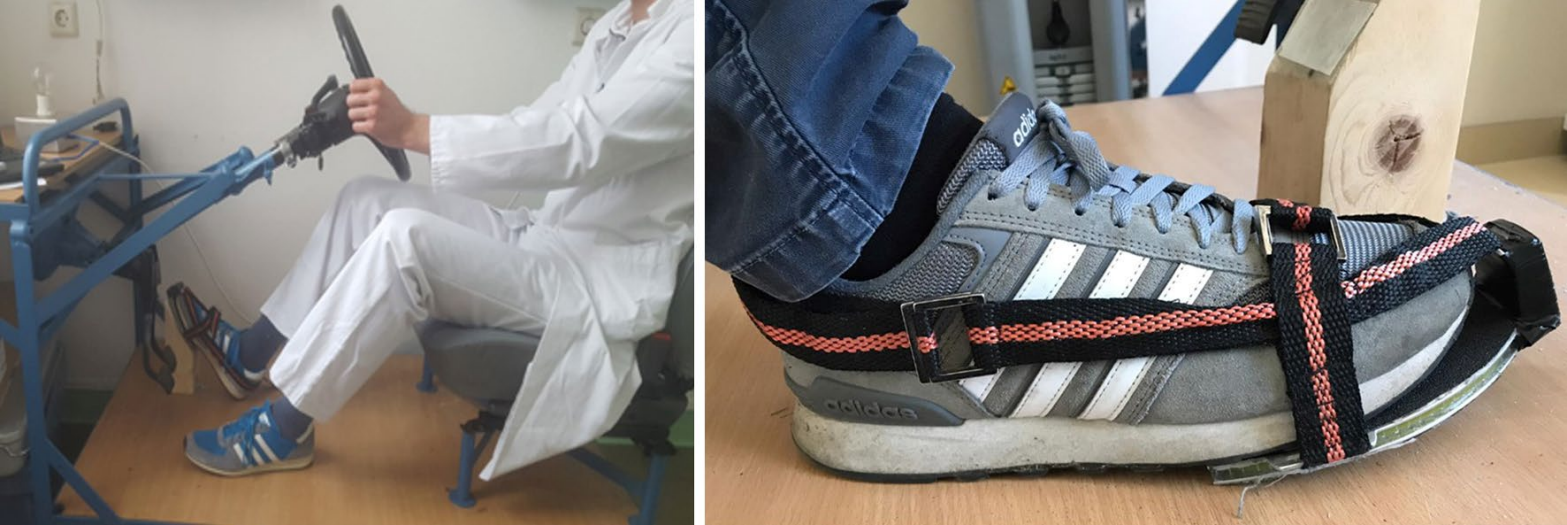
Driving simulator: the image on the left shows the cockpit of the driving simulator; the image on the right shows the measurement template sole with an integrated data transmitter

**Fig. 3:**
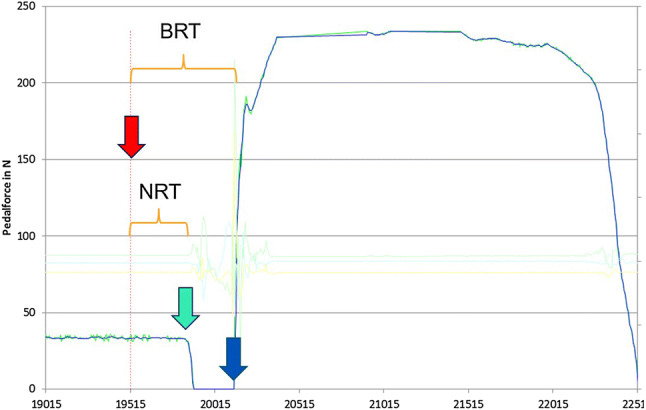
Depiction of an exemplary measurement curve: the blue curve shows the force acting on the measurement template in Newton [N]; the pink arrow marks random activation of the signal lamp; the green arrow indicates release of the right foot from the accelerator pedal and hence initiation of the braking process; the blue arrow indicates the start of brake pedal actuation; neuronal response time (NRT) denotes the period from activation of the signal lamp to release of the accelerator pedal; brake response time (BRT) denotes the period from activation of the signal lamp to actuation of brake pedal; brake pedal force (BPF) denotes the maximum deflection of the measurement curve (blue)

The employed car cockpit simulator was set up in cooperation with an external service provider (Automobilservice Fröde, Berlin, Germany, Fig. 2) based on previously described simulators [14, 20]. The measuring electronics and processing software were provided by an external company (software “Pedal Force Measurement“, FSD Fahrzeugsystemdaten GmbH, Dresden, Germany). The pedal force metre used the hydraulic measuring principle and recorded pedal movements and system pressure at every 5 ms. The employed software displays these variables in the form of a measurement curve as well as corresponding Microsoft Excel table and allows identification of exact BPF in Newton [N] over time (Fig. 3).

Measurements were performed based on a standardised protocol: Patients had to initiate an emergency stop in response to an external optical signal (flash of signal lamp) that was triggered by a random number generator. During the simulation, subjects had to move their foot from the accelerator pedal to the brake pedal after noticing the optical signal. To evaluate reliability of our measurements, two independent measurements were taken for each study participant in the control group, with intervals of at least 10 min between the individual braking processes. Measurements in the patient group were performed prior to surgery (mean 1.0 days ± 0 [0]), postoperatively at hospital discharge (mean 5.5 days ± 1 [4–9]), during second half of in-hospital rehabilitation (15th–31st postoperative days) (mean 23.9 days ± 5.5 [15–31]) and after finishing in-hospital rehabilitation (32–58 postoperative days) (mean 42.7 days ± 7 [32–58]) after TKR. The given ranges of measurement appointments were due to organisational reasons.

Reaction times and BPF were recorded at every time point. NRT [ms] (NRT) quantifies the amount of time from illumination of the signal lamp to releasing the accelerator pedal and is, therefore, a component of BRT [ms] [1]. BRT is the amount of time needed from the beginning of the signal stimulus to actuation of the brake pedal [15, 19]. BPF describes the maximum force exerted on the brake pedal [17]. Additionally, we documented epidemiological data (height, weight, age, sex), driving frequency, pain [scored with a numerical rating scale (NRS)], subjective fitness to drive (scored according to the German school grading system with 1 being the best and 6 being the worst), and the duration of forearm crutch use. Forearm crutches were reportedly used in everyday life for an average of 5.8 ± 3.1 weeks [2–12].

This prospective cohort study was conducted between 2017 and 2018 with the approval by the institutional review board (Charité University Hospital Berlin, EA1/143/16).

### Statistical evaluation

A priori power analysis was conducted by our statistic consultant to estimate the sample size required to test for significant differences based on the reported BPF according to Raudszus et al. [17]. For a significance level of 5%, a power of 80%, and a non-inferiority margin of 130 N between patient and control group, a minimum of twenty-seven subjects were needed for each group. Nquery 7.0 software was used to calculate the number of cases needed. All statistical calculations were performed with SPSS Version 26 (SPSS Inc., Chicago, IL, USA).

Test–retest reliability was assessed using Wilcoxon side rank test on the two independent braking manoeuvres of the control group.

For normal distributed data, student’s *t* test was employed to test for significance in change over time for all assessed parameters in the TKR group. In all other cases, Wilcoxon side rank test was used to calculate significance. Additionally, we tested for significant differences between BPF of the TKR group and the control group for each time point using either the unpaired *t* test or the Mann–Whitney *U* test, depending on normal distribution. A *p* < 0.05 was defined as statistically significant.

Additionally, Pearson correlation test was performed comparing NRT, BRT and BPF with the exact number of postoperative days within the 3rd and 4th measurement appointments in order to exclude any impact of chosen time ranges mentioned above. We also performed a Pearson correlation test on all subjects to determine a potential influence of age on NRT, BRT, and BPF.

## Results

### Evaluation of the braking process in the control group

NRT, BRT and BPF showed considerable inter-individual but no intra-individual differences demonstrating test–retest reliability (Table 1).

**Table 1:** Results of the control group measurements

	NRT (ms)	BRT (ms)	BPF (N)
**Measurement 1**			
Mean	**457.8 ± 186.1**	**712.7 ± 160.6**	**649.3 ± 245.1**
Range	*239–1394*	*475–1304*	*305–1337*
**Measurement 2**			
Mean	**443.2 ± 88.1**	**698.5 ± 127.5**	**648.7 ± 231.3**
Range	*302–735*	*490–962*	*295–1283*
Significance	*p* = 0.417^n.s.^	*p* = 0.874^n.s.^	*p* = 0.572^n.s.^
**Average**			
Mean	***450.5 ± 122.5***	***705.6 ± 119.8***	***648.9 ± 213.5***
Range	*306–982*	*512–973*	*320–1057*
95% CI	*413.7/487.3*	*669.6/741.6*	*584.8/713.1*

Bolditalic values represent the average of all measured parameters in control group used for further comparisons and reference

*NRT* neuronal reaction time, *BRT* brake reaction time, *BPF* brake pedal force, *CI* confidence interval, *n.s.* not significant

Regarding patients’ gender, no significant differences in NRT (women 460.6 ms ± 107.7 [348–900], men 436.8 ms ± 142.2 [306–982], *p* = 0.1) or BPF (women 601.1 N ± 183.5 [320–1007], men 714.6 N ± 238.3 [360–1057], *p* = 0.078) were found. BRT was significantly lower in male subjects (women 749.8 ms ± 120.3 [525–973] vs. men 645.1 ms ± 91.1 [512–793], *p* = 0.003).

### Evaluation of subjective outcome parameters in the patient group

Mean knee joint pain was rated 5.1 ± 2.3 [1–9] preoperatively, 4.9 ± 2.1 [2–9] immediately after surgery, 2.9 ± 1.7 at postoperative week 3/4 [1–7], and 2.1 ± 1.3 [1–6] at postoperative week 6. Pain was significantly reduced starting from week 3/4 (*p* < 0.001).

**Fig. 4:**
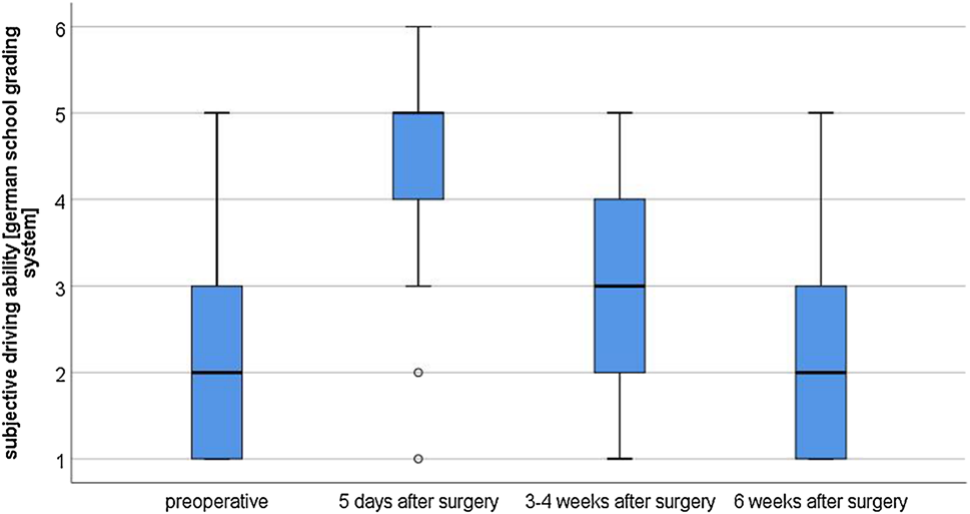
Subjective driving ability; rated according to the German school grading system (1-“very good”, 6 “insufficient”). Prior to surgery, patients rated their driving ability “good” (2.3 ± 1.2) [1–5]. Subjective ratings decreased to 4.4 ± 1.2 after surgery [1–6]. Driving ability was rated 2.9 ± 1.4 [1–5] in week 3/4 and 2.2 ± 1.2 [1–5] in week 6. Only comparison between the 1st and 2nd measurements showed a significant subjective impairment (*p* < 0.001). Measurements from the 3rd/4th week and the 6th week were no longer significantly decreased (*p* = 0.096 and *p* = 0.514)

Regarding the impact of their right knee joint symptoms on their driving ability, patients reported the restoration of a “good” driving experience at week 6 on average (Fig. 4).

### Evaluation of the braking process in the patient group

In detailed comparison of NRT, BRT and BPF between preoperative and postoperative time points, only BPF showed a significant reduction during the early postoperative period (*p* < 0.001) (Tables 2, 3).

**Table 2:** Evaluation of the braking process in the patient group

	Preoperative	5 days after operation	3–4 weeks after operation	6 weeks after operation
**NRT (ms)**				
Mean	**505.7 ± 162.1**	**560.5 ± 218.1**	**581.0 ± 163.8**	**546.1 ± 127.3**
Range	*336–1250*	*344–1576*	*302–1028*	*351–899*
95% CI	*455.2/566.2*	*479.1/641.9*	*529.9/642.1*	*498.6/593.7*
**BRT (ms)**				
Mean	**984.7 ± 356**	**1090.0 ± 404.2**	**1013.1 ± 260.1**	**934.1 ± 233.1**
Range	*567–2115*	*513–2602*	*460–1642*	*589–1553*
95% CI	*851.8/1117.5*	*940.0/1241.9*	*916.0/1110.3*	*847.1/1021.2*
**BPF (N)**				
Mean	**613.5 ± 291.5**	**411.0 ± 190.6**	**554.7 ± 199.6**	**658.9 ± 308.9**
Range	*278–1638*	*157–890*	*283–1169*	*194–1715*
95% CI	*504.6/722.3*	*339.8/482.2*	*480.2/629.2*	*543.6/774.3*

*NRT* neuronal reaction time, *BRT* brake reaction time, *BPF* brake pedal force, *CI* confidence interval

**Table 3:** Detailed comparison of significance level of NRT, BRT and BPF

Measurement	NRT	BRT	BPF
Preoperative vs.			
5 days after operation	0.253^n.s.^	0.086^n.s.^	< 0.001*
3–4 weeks after operation	0.095^n.s.^	0.478^n.s.^	0.081^n.s.^
6 weeks after operation	0.276^n.s.^	0.781^n.s.^	0.07^n.s.^

*NRT* neuronal reaction time, *BRT* brake reaction time, *BPF* brake pedal force, *n.s*. not significant

*Significant

To rule out a potential impact of variations in follow-up times, Pearson correlation test was performed for NRT, BRT, and BPF versus the exact number of postoperative days. No significant correlation was found for any of the tested parameters (3rd/4th-week correlation: NRT 0.196, *p* = 0.299; BRT − 0.078, *p* = 0.688; BPF 0.154, *p* = 0.416; 6th-week correlation: NRT − 0.081, *p* = 0.67, BRT − 0.217, *p* = 0.25, BPF − 0.217, *p* = 0.25).

### Comparison of the control and patient groups

Only (preoperative) BPF showed no significant differences between patient and control group (Table 4).

**Table 4:** Comparison between control and preoperative patient group data

	Control group	Patient group	Significance
**NRT (ms)**			
Mean	**450.5 ± 122.5**	**509.8 ± 163.6**	*p* = 0.011*
Range	*306–982*	*336–1250*	
95% CI	*413.7/487.3*	*448.8/570.9*	
**BRT (ms)**			
Mean	**705.6 ± 119.8**	**984.7 ± 355.8**	*p* < 0.001*
Range	*512–973*	*567–2115*	
95% CI	*669.6/741.6*	*851.8/1117.5*	
**BPF (N)**			
Mean	**648.9 ± 213.5**	**609.6 ± 296**	*p* = 0.245^n.s.^
Range	*320–1057*	*194–1638*	
95% CI	*584.8/713.1*	*498.9/720.3*	

*NRT* neuronal reaction time, *BRT* brake reaction time, *BPF* brake pedal force, *CI* confidence interval, *n.s.* not significant

*Significant

A significant correlation was identified between age and NRT (Pearson 0.251, *p* = 0.03), as well as BRT (Pearson 0.536, *p* < 0.001). There was no correlation between age and BPF (Pearson − 0.13, *p* = 0.268).

As merely BPF showed a significant decrease in the patient group after TKR, a detailed comparison between the control and patient groups was performed for BPF only (Table 5).

**Table 5:** Comparison of BPF after surgery between control and patient group

Timepoint	Parameter in mean + Standard deviation	Control group	Significance [95% CI]
5 days after surgery	411.0 N ± 190.6 *[157–890]*	648.9 N ± 213.5 *[320–1057]*	< 0.001* *[141.8 N, 334.2 N]*
3–4 weeks after surgery	554.7 N ± 199.6 *[283–1169]*		0.058^n.s.^ *[− 3.5 N, 192.1 N]*
6 weeks after surgery	658.9 N ± 308.9 *[194–1715]*		0.869^n.s.^*[− 130.1 N, 110.2 N]*

*BPF* brake pedal force, *CI* confidence interval, *n.s.* not significant

*Significant

## Discussion

The most important finding of the study was that only BPF showed a significant decrease after TKR and preoperative baseline levels were only reached 6 weeks after surgery. In addition, subjective outcome parameters did not show satisfying results until 6 weeks after surgery. These results suggest that a timeframe of 6 weeks after TKR should be kept before patients can safely start driving again confirming our hypothesis.

A majority of patients view rapid restoration of the postoperative driving ability as a key issue due to its necessity for mobility and independence. The ability to perform a quick and effective emergency stop is essential for the safe operation of a motor vehicle. The heterogeneity of the published data illustrates the difficulties encountered when trying to define an appropriate recommendation with due consideration of individual circumstances. This study, therefore, aimed to assess driving ability as defined by NRT, BRT, and BPF as well as subjective outcome parameters. Measurements were taken at different time points over the perioperative period to evaluate different stages in the restoration of a patients driving ability following TKR.

NRT, BRT, and BPF exhibited a large inter-individual range in the control group. BRT in younger (32 years) control subjects (706 ms) was similar to values previously reported (700–750 ms) [6]. In contrast, the older patient group (66 years) exhibited significantly slower (985 ms) preoperative reaction times.

Of note, we found braking response times to be faster for the male control population. As NRT measurements were similar, the cause seemed to be a shorter transfer time from the accelerator to the brake pedal. Similarly, Green and Li et al. found women to have a slower brake reaction time (BRT) in critical situations [6, 10]. In contrast to our results, Li and colleagues found that women responding to an emergency signal while operating a mobile phone responded slower than men, but ended up with a shorter braking distance due to more efficient braking [10]. Other studies, however, failed to show any significant gender differences regarding BRT [1, 5]. In summary, the available evidence does not provide clear evidence for gender-specific recommendations regarding driving safety.

We also found a correlation between age and reaction time, although the comparison is limited as the majority of older patients complained of severe knee pain. Besides pain, restriction of movement due to advanced gonarthrosis may be a decisive factor for the found age-dependent differences. Other authors have confirmed this observation [2, 4]. In contrast, Dalury et al. described a significantly shorter preoperative BRT of 530 ms in patients with gonarthrosis; while, Hernandez et al. observed a value of 692 ms [1, 7]. On a critical note, comparison of reaction times is complicated by great variances (430 ms–1330 ms) due to the heterogeneity of patient groups and the simulators employed [1, 7, 8, 11, 12]. Thus, driving ability recommendations based on NRT or BRT only might be prone to error and insufficient in many cases. As discussed by McLeod et al., evaluating BPF is critical to make reliable recommendations [13]. In contrast to the reaction time results, this study did not determine any significant differences in BPF between genders or between the healthy control group and the preoperative patient cohort. Hence, preoperative chronic knee pain (NRS 5.1) does not seem to be associated with any significant reduction in BPF. No study so far has described influential factors or a correlation between BPF and age. In conclusion, BPF measurements offer decisive advantage for the evaluation of driving ability, especially after surgical intervention.

Both reaction time measurements and BPF reported in literature show pronounced inter-individual fluctuations, complicating establishment of universally applicable quantitative thresholds. With regard to assessing driving abilities, priority should be given to considering the individual preoperative baseline parameters and not to obtain arbitrarily defined thresholds.

Overall, NRT increased by 55 ms and BRT by 105 ms immediately after surgery. Despite the absence of statistical significance, the latter is equivalent to an extension of the braking distance by approximately 1.5 m at a speed of 50 km/h and to an increase of approximately 3 m at a speed of 100 km/h. Jordan at al. observed a similar non-significant prolongation of the BRT by 150 ms at 8 days after lower limb surgery [9]. Other authors also found no significant prolongation of BRT during the postoperative period [14]. On the other hand, a recent meta-analysis by Van der Velden et al. identified nine prospective studies providing partly inhomogeneous recommendations based solely on changes in reaction time [21]. Pooling all data after right-sided TKR, reaction time reached preoperative baseline levels 4 weeks after TKR. However, none of these studies evaluated braking force as an additional outcome parameter. Another systematic review by Di Silvestro et al. concluded that operation of a motor vehicle is possible 4 weeks after TKR based on changes in reaction time [3]. However, only 4 of the 25 investigated reports considered braking force for their recommendations (10 days to 12 weeks), and none of them focused on TKR patients [3]. To our best knowledge, only Spalding et al. evaluated BPF as an additional safety parameter after TKR [18]. However, this study is 26-years old and is therefore not taking current technical developments such as fast track concepts and modern rehabilitation protocols into account.

BPF was found to be significantly decreased immediately after surgery. Although no statistically significant difference was detected in the 3rd–4th postoperative weeks compared to the preoperative levels, baseline values were only fully restored after the 6th postoperative week. Furthermore, no significant difference between control and patient group was observed in week 3/4 after surgery. At present, there is paucity of literature concerning braking force after total joint replacement [13]. Jordan et al. investigated BPF over time (preoperatively, 8 days, 6, 12, and 52 weeks postoperatively) after hip replacement [9]: for these patients, BPF was significantly reduced and did not return to preoperative levels until week 12. In contrast, the BRT had already been restored in the 6th postoperative week. The prolonged convalescence in the Jordon et al. study when compared to the results presented in our study may be due to postoperative rehabilitation differences between knee and hip replacements, as well as mobility restrictions relating specifically to the hip.

In addition to the objective evaluation of driving ability by measuring NRT, BRT and BPF, subjective parameters like pain levels or subjective fitness to drive are key parameters in the decision-making process. Regardless of the statistical significance of the physical parameters assessed in this study, patients did not rate their own driving ability to be “good” until the 6th postoperative week. Moreover, crutches were used postoperatively for approximately 5–6 weeks. Current german case-law interprets the use of walking aids as negligent behaviour and could therefore attribute to (partial) liability in the event of a traffic accident, regardless of an individual’s subjective physical fitness (§ 315c of the German Criminal Code, StGB). Taking into account all subjective and objective parameters, patients’ driving ability was restored to preoperative levels 6 weeks after surgery. These results suggest that active participation in road traffic should generally not take place before the 6th postoperative week.

Some limitations apply to this study. The control group was not matched for age or gender. It was, therefore, not possible to reliably evaluate the impact of age and pain on NRT, BRT, and BPF. Nonetheless, comparison with preoperative baseline values appears sufficient due to the described inter-individual fluctuations. Furthermore, comparing patients to a healthy younger control group results in an even stricter evaluation. The authors are also aware that a time range of approximately 14 days for the 3rd and 4th measurements might impact the braking behaviour. Due to patient-initiated changes of scheduled appointments and organisational reasons, it was not possible to assess all patients at the exact same day after surgery. However, as rehabilitation and function improve only slowly over the postoperative course, the authors are confident that this does not result in a substantial bias of the data. Furthermore, correlation analysis failed to show any influence of time ranges within the separate measurement timepoint. A third limitation is that the effects of brake boosters on emergency braking were not considered. This study was not able to evaluate the extent to which a modern brake booster might achieve adequate braking, even with reduced BPF. By definition, however, emergency braking requires a rapid and powerful braking manoeuvre.

## Conclusion

Because of significant inter-individual fluctuations, a comparison between pre- and postoperative parameters seems more reliable than defining absolute thresholds. Taken together, all objective and subjective parameters indicate that patients do not regain preoperative driving ability levels until postoperative week 6. Hence, active participation in road traffic should not be recommended until the 6th week following surgery.

## Abbreviations

TKR: Total knee replacement
BPF: Brake pedal force
NRT: Neuronal reaction time
NRS: Numeric Rating Scale
BRT: Brake reaction time
